# Kidney-sparing management of a giant distal ureteric stone: A case report

**DOI:** 10.1016/j.ijscr.2025.111964

**Published:** 2025-09-20

**Authors:** Mahmoud Mustafa, Amir Aghbar, Ibraheem Alami, Razi Sulaiman, Mohammad Khader

**Affiliations:** a Department of Medicine, Faculty of Medicine and Allied medical Sciences, An-Najah National University, Nablus, 44839, Palestine; b Department of Urology, An-Najah National University Hospital, Nablus, 44839, Palestine; c Department of Radiology, An-Najah National University Hospital, Nablus, 44839, Palestine

**Keywords:** Giant distal ureteric stone, Hydronephrosis, Kidney-preserving strategy, Laser lithotripsy, Balloon ureteroplasty

## Abstract

**Introduction and importance:**

Giant distal ureteric stones are rare and pose significant treatment challenges due to their size and associated risk of renal impairment. While nephrectomy is often considered, kidney-sparing strategies may offer functional preservation, especially when aligned with patient preferences.

**Presentation of case:**

A 75-year-old male with comorbid hypertension and gout was found to have a 5.6 × 2.8 cm distal ureteric stone causing severe hydronephrosis and cortical thinning. Despite initial consideration for nephrectomy, the patient opted for a kidney-preserving approach. He underwent staged interventions, including ureteroscopy with laser lithotripsy, nephrostomy insertion, balloon ureteroplasty, and antegrade DJ stent placement. Complete stone clearance and resolution of obstruction were ultimately achieved.

**Clinical discussion:**

This case highlights the complexity of managing large ureteric stones and the value of a multimodal endourological approach in challenging anatomy. The use of balloon ureteroplasty and antegrade stenting was critical in overcoming obstruction after failed wire passage. The case also reflects the importance of respecting patient autonomy while adhering to guideline-based management.

**Conclusion:**

A personalized, minimally invasive, kidney-sparing strategy can achieve successful outcomes in cases of giant distal ureteric stones, even when initial imaging suggests poor renal prognosis.

## Highlights

• Giant 5.6 cm distal ureteric stone managed successfully without nephrectomy.
• Multimodal kidney-sparing strategy resolved obstruction and preserved renal function.
• Stepwise laser lithotripsy and balloon ureteroplasty ensured complete stone clearance.
• Severe hydronephrosis reversed using endourological tools and nephrostomy drainage.
• Case illustrates guideline-based, patient-centered care for complex ureteric stones.

## 1 Introduction

Ureteric stones are a common cause of urinary tract obstruction [1], with large stones in the distal ureter posing significant challenges to both stone clearance and preservation of renal function [2]. Stones larger than 1 cm are unlikely to pass spontaneously and typically require surgical intervention. The EAU guidelines do not provide an exact cutoff for stone size, describing them simply as “small.” They emphasize, however, that the likelihood of spontaneous passage decreases as the stone becomes larger and varies individually among patients [3]. In contrast, the AUA guidelines clearly define the applicable stone size for this approach as ≤10 mm [[4], [5], [6]]. Delayed or inadequate treatment of such stones can result in severe complications, including hydronephrosis, cortical thinning, and irreversible kidney damage [7]. Management options for large stones often include a combination of ureteroscopy, laser lithotripsy, and adjunctive procedures like stenting or balloon dilation, particularly when standard methods are insufficient [1,5,8]. This case report illustrates the complexity involved in managing a large distal ureteric stone, emphasizing the need for a tailored, stepwise strategy to overcome the difficulties associated with such challenging cases. This case report has been reported in line with the SCARE checklist [9].

## 2 Case presentation

A 75-year-old male patient, known for hypertension and gout that is manage with Amlodipine and Allopurinol, initially presented with lower limb pain. There was no known family history of nephrolithiasis or hereditary renal disease. The patient is a retired teacher, non-smoker, and reported adequate fluid intake. No history of excessive dietary oxalate, animal protein, or sodium consumption was noted.

**Fig. 1:**
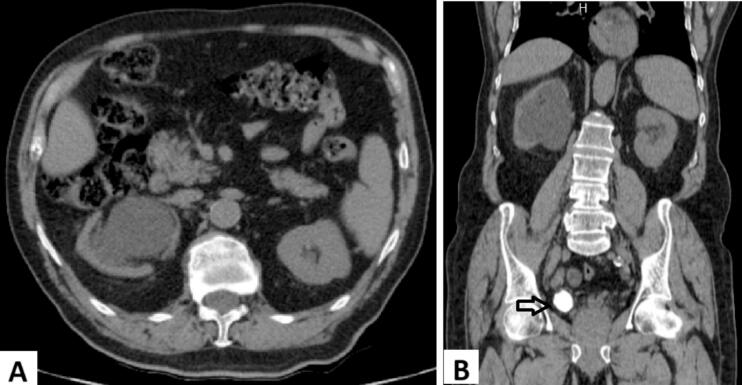
Preoperative computed tomography (CT) scan demonstrating severe right sided hydronephrosis with thinning of the right renal cortex. (A) Axial CT view, (B) coronal CT view. Arrow in (B) pointing to the large right distal ureteral stone.

**Fig. 2:**
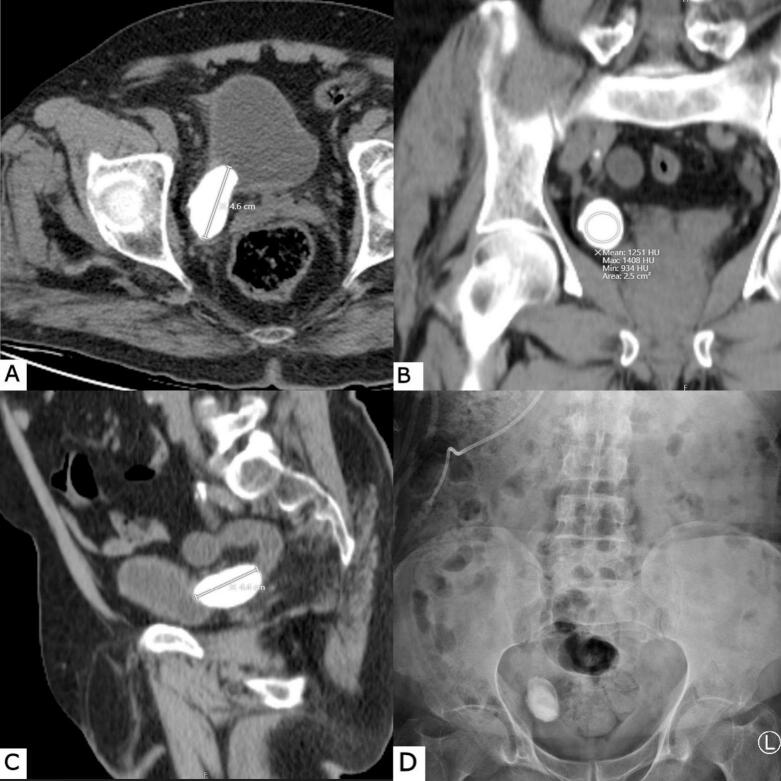
Preoperative computed tomography (CT) scan demonstrating a 5.6 ∗ 2.8 right distal ureteric stone, and with Hounsfield Unit values ranging from 934 to 1430. (A) Axial CT view, (B) coronal CT view, and (C) sagittal CT view. (D) A plain KUB X-ray confirms the radiopaque stone in the right distal ureter.

During the evaluation for gout, elevated serum creatinine level was detected (1.66 mg/dl). He denied any history of flank or abdominal pain, hematuria, or previous urinary stones. A renal CT scan without contrast revealed severe right hydronephrosis and hydroureter (Fig. 1) due to a distal ureteric stone measuring 5.6*2.8 cm, with a density of 1250 HU, resulting in thinning of the cortex (Fig. 2). No other stones were observed along the urinary tract.

**Fig. 3:**
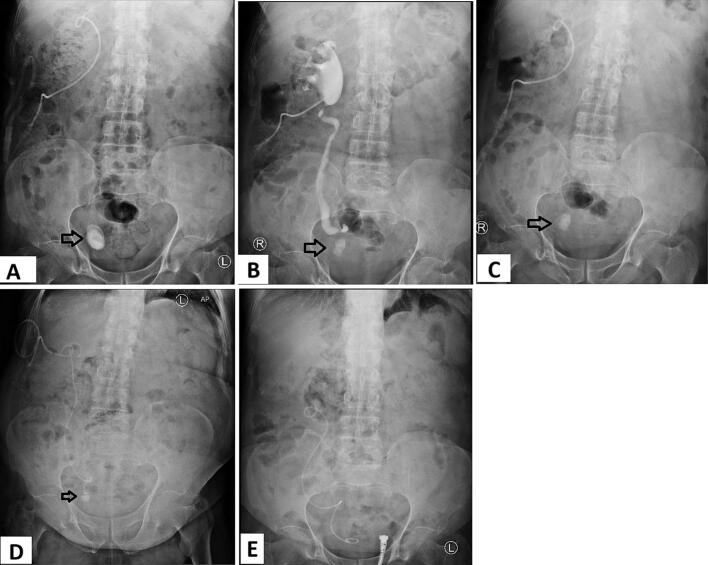
Chronologically ordered KUB X-rays. (A) After the first ureteroscopy and laser lithotripsy and showing right nephrostomy placed. (B) Antegrade nephrostogram showing a significantly dilated right pelvicalyceal system and right ureter with the distal ureter obstructed by the stone. (C) After the second ureteroscopy and laser lithotripsy, (D) showing right nephrostomy and antegradely inserted DJ stent in place, with the tip of the stent abutting the residual stone fragment. (E) Final image after complete fragmentation and clearance of the stone and fragments, and its shows the right DJ stent in place, being distally in the urinary bladder, and proximally in the dilated upper ureter. Arrow pointing to the right distal ureteral stone.

Given the loss of renal parenchyma on the initial CT scan (Fig. 1) and strong indications of a poorly functioning kidney, the options of undergoing a renal scintigraphy scan and nephrectomy were discussed with the patient. In addition, open and laparoscopic ureterolithotomy approaches were explained to the patient. However, he refused all options, expressing a preference for a kidney-preserving approach. Thus. the patient underwent initially right ureteroscopy and laser lithotripsy of the stone. However, only about 50 % of the stone was fragmented, and attempts to insert a wire proximal to the impaction site failed. Consequently, a right nephrostomy tube was inserted. Despite the placement of the nephrostomy tube, the output did not exceed 500 cc per day (Fig. 3-A).

The main challenging anatomy in this case was to access the ureter above the stone in the first session of fragmentation. This was due to the large burden of stone fragments and the tortuous dislocation of the ureter above the stone.

Approximately one month later, the patient underwent another ureteroscopy session with laser lithotripsy. Antegrade pyelography revealed a significantly dilated right renal pelvis and a dilated and tortuous right ureter proximal to the stone (Fig. 3-B). Retrograde study indicated no contrast passage above the stone, suggestive of complete right distal ureter obstruction. The stone was completely fragmented during the procedure, but inserting a sensor wire into the proximal right ureter was unsuccessful (Fig. 3-C).

After another month, the patient underwent balloon ureteroplasty of the distal ureter with antegrade ureteric DJ stent insertion and nephrostomy replacement (Fig. 3-D). Balloon dilation was performed by the interventional radiologist during antegrade stenting. The aim was to facilitate passage of the sensor wire and access to the ureter, rather than to treat a stricture. A follow up CT scan showed a dilated and tortuous right ureter, dilated right renal pelvis, multiple stone fragments in the distal ureter, DJ stent with its distal end at the site of stone fragments, and the right nephrostomy in place.

Two months later, the patient presented for DJ stent replacement. During the procedure, all stone fragments in the right distal ureter were withdrawn to the urinary bladder using forceps and a basket, leaving no fragments behind (Fig. 3-E). Retrograde pyelography demonstrated a dilated right ureter, significantly dilated right renal pelvis, and right pelvicalyceal system. The DJ stent was removed after one month.

Overall, the patient underwent a series of staged interventions, over 5 months period, to address complications arising from a large distal ureteric stone, including ureteroscopy, ureteroplasty, stent insertion, and nephrostomy, ultimately achieving successful stone clearance and resolution of obstruction. The patient expressed gratitude for the kidney-sparing approach and was satisfied with the outcome despite the prolonged treatment. Serum creatinine fluctuated between 1.66 and 2.15 mg/dl during and after the procedures.

The procedures were performed at a tertiary-level urology center with a high volume of endourological interventions. All interventions were conducted by a consultant urologist with over 30 years of experience in endourology, assisted by a senior urology resident with more than four years of specialty training. Rigid ureteroscopy (Karl Storz, 7.5 Fr, 34 cm length) was used during all ureteroscopic interventions. Moreover, a consultant interventional radiologist was responsible for percutaneous and antegrade approach to the kidney including antegrade ureteroplasty. Procedures were performed in the operation room under complete sterile techniques. No urinary tract infections were detected on urinalysis or urine culture performed prior to each endourological intervention, and the patient did not develop any symptomatic UTI following any of the procedures.

## 3 Discussion

The AUA and EAU guidelines discuss the management of large ureteric stones with a focus on stone clearance and preservation of renal function; however, their recommendations are not always explicit, and interpretation often depends on the clinical context [5,8]. In this case, the 5.6 × 2.8 cm stone in the distal ureter posed a significant risk of irreversible renal damage, as indicated by severe hydronephrosis and cortical thinning. According to both sets of guidelines, stones larger than 1.5 cm in the ureter are unlikely to pass spontaneously and typically require ureteroscopy or other surgical intervention [5,8]. For stones greater than 2 cm, a multimodal approach involving ureteroscopy and percutaneous nephrostomy, followed by staged procedures, is often required to achieve complete clearance [7].

Giant ureteric stones have been defined in the literature as stone having a size greater than 5 cm, and few have been described in the literature [10]. Initial management with ureteroscopy and laser lithotripsy resulted in partial fragmentation, but as described in the EAU guidelines, larger stones may necessitate multiple sessions for complete removal [8]. The inability to pass a wire beyond the impacted stone, compounded by the tortuosity and dilation of the ureter, further complicated this case. Both the AUA and EAU guidelines acknowledge these challenges, recommending adjunctive procedures such as balloon ureteroplasty or stenting, as employed in this patient [5,8]. Laparoscopic ureterolithotomy is also a feasible option to consider [10].

The patient's refusal of nephrectomy, despite the evidence of poor renal function, illustrates the balance between adhering to guidelines and respecting patient autonomy. While nephrectomy would have been a reasonable option according to AUA guidelines for a poorly functioning kidney with significant obstruction [5], the decision to pursue a kidney-preserving approach aligned with the EAU's emphasis on individualized care [8], considering patient preferences and quality of life.

In line with the guidelines, nephrostomy placement and subsequent balloon ureteroplasty helped alleviate obstruction, but the persistence of hydronephrosis underscores the complexity of managing such cases. The eventual successful clearance of stone fragments and removal of the stent marked the resolution of the obstruction, although the patient required prolonged care over multiple procedures.

The strength of this case report lies in the successful management of a rare giant distal ureteric stone using a minimally invasive, kidney-sparing approach tailored to the patient's preferences. It demonstrates the value of a stepwise endourological strategy in complex anatomy. However, as a single case, its generalizability is limited, and long-term renal function was not objectively assessed through follow-up scintigraphy. Future studies are needed to assess long-term outcomes and guide standardized management of giant ureteric stones.

In conclusion, this case exemplifies the challenges outlined by both AUA and EAU guidelines [3,5,8] in managing large ureteric stones, particularly in cases with significant obstruction and renal impairment. The combination of endourological techniques, including ureteroscopy, stenting, and balloon ureteroplasty, ultimately led to a successful outcome, though the prolonged course of treatment highlights the complexity of such cases. Respect for patient autonomy remains a critical component of management, even when guidelines suggest alternative approaches.

## Author contribution

• **Mahmoud Mustafa**: Conceptualization, Surgical management, Writing – original draft, Resources.
• **Amir Aghbar**: Surgical management, Data curation, Writing – review & editing, Visualization, Supervision.
• **Ibraheem Alami**: Data acquisition, Literature review, Writing – original draft.
• **Razi Sulaiman**: Data acquisition, Patient follow-up, Investigation, Writing – review & editing.
• **Mohammad Khader**: Radiological analysis and management, Validation, Critical revision of the manuscript, Final approval.

All authors have read and approved the final manuscript.

## Consent

A written informed consent was obtained from the patient for the purpose of this article publication and all related images. A copy of the written consent is available for review by the Editor-in-Chief of this journal on request.

## Guarantor

Amir Aghbar.

## Research registration number

Not applicable.

## Funding

None.

## Conflict of interest statement

No conflict of interest to declare.

## References

[1] Bosshard P., Stritt K., Roth B. (2020). Overview of ureteral stone management. Rev. Med. Suisse.

[2] Okçelik S., Kurul N.O., Kiziloz H., Temel M.C., Yesildal C. (2021). Factors affecting success of semi-rigid ureterorenoscopy in proximal ureter stone treatment. J. Coll. Physicians Surg. Pak..

[3] Skolarikos HJ A., Neisius A., Petřík A., Kamphuis G.M., Davis N.F., Somani B., Tailly T., Lardas M., Gambaro G., Sayer J.A., Geraghty R., Lombardo R., Tzelves L., Bezuidenhout C. (2025). EAU guidelines on Urolithiasis the EAU Annual Congress Madrid 2025.

[4] Pearle M.S., Goldfarb D.S., Assimos D.G., Curhan G., Denu-Ciocca C.J., Matlaga B.R. (2014). Medical management of kidney stones: AUA guideline. J. Urol..

[5] Assimos D., Krambeck A., Miller N.L., Monga M., Murad M.H., Nelson C.P. (2016). Surgical management of stones: American urological association/endourological society guideline, PART I. J. Urol..

[6] Akram M., Jahrreiss V., Skolarikos A., Geraghty R., Tzelves L., Emilliani E. (2024). Urological guidelines for kidney stones: overview and comprehensive update. J. Clin. Med..

[7] Wein A.J.K.L., Partin A.W., Peters C.A., Dmochowski R.R. (2020). Campbell-Walsh-Wein Urology.

[8] Türk C., Petřík A., Sarica K., Seitz C., Skolarikos A., Straub M., Knoll T. (2016). EAU guidelines on interventional treatment for urolithiasis. Eur. Urol..

[9] Ahmed K., Ahmed A., Ginimol M., Catrin S., Rasha R., Thomas F. (2025). Revised Surgical CAse REport (SCARE) guideline: an update for the age of Artificial Intelligence. Premier Journal of Science..

[10] Rathod R., Bansal P., Gutta S. (2013). A giant ureteric calculus. Indian J. Urol..

